# Serpin Family A Member 1 Is Prognostic and Involved in Immunological Regulation in Human Cancers

**DOI:** 10.3390/ijms241411566

**Published:** 2023-07-17

**Authors:** Xingwang Kuai, Jiaying Lv, Junyu Zhang, Manyu Xu, Juling Ji

**Affiliations:** 1 Department of Pathology, Department of Clinical Biobank, Medical School of Nantong University, Nantong 226001, China; 2113510016@stmail.ntu.edu.cn (X.K.); 2113310028@stmail.ntu.edu.cn (J.L.); 2113310034@stmail.ntu.edu.cn (J.Z.); 2 Department of Clinical Biobank, Affiliated Hospital of Nantong University, Nantong 226001, China; 2013310036@stmail.ntu.edu.cn

**Keywords:** SERPINA1, prognosis, immune infiltration, immunotherapy, pan-cancer

## Abstract

Serpin family A member 1 (SERPINA1) encodes a protease inhibitor participating in many human diseases, but its value in immunoregulation and prognosis of human cancers remains unclear. In this study, through comprehensive analysis of data from The Cancer Genome Atlas (TCGA) datasets, we found that SERPINA1 was dysregulated in many cancers compared with normal tissues. SERPINA1 expression was significantly associated with prognosis, immune subtype, molecular subtype, immune checkpoint (ICP) genes, tumor mutational burden (TMB), microsatellite instability (MSI), and the estimation of stromal and immune cells in malignant tumor tissues using expression data (ESTIMATE) score. There was a strong connection between SERPINA1 expression and tumor-infiltrating lymphocytes, and SERPINA1 showed significant relation to gene markers of immune cells in digestive tumors. Fluorescence-based multiplex immunohistochemistry confirmed that SERPINA1 protein expression was related to clinicopathologic features and immune infiltrates in hepatic cancer. This study suggests that SERPINA can potentially serve as a novel biomarker for cancer prognosis and immunotherapy.

## 1. Introduction

Immune-based therapies have revolutionized the treatment of advanced cancer. The application of immune checkpoint inhibitor therapy targeting the PD-1/PD-L1 or CTLA-4 pathway is a breakthrough for many types of cancer [1]. The tumor microenvironment (TME) comprises tumor cells, immune cells, stromal cells, intricate cytokines, and chemokines, providing a favorable environment for tumor growth [2]. Tumor cells secrete growth factors and cytokine and regulate interstitial and immune cells’ behaviors. The interaction between tumor cells and the TME influences the response to immunotherapy [3]. Therefore, it is necessary to identify novel prognostic biomarkers of immunotherapy.

*Serpin family A member 1* (*SERPINA1*) is located on chromosome 14 (14q32.1), encoding the protein Alpha1-antitrypsin (A1AT) that is a highly conserved protein and the dominant protease inhibitor, the inhibition capacity of which accounts for more than 90% of all plasma proteases [4]. *SERPINA1* is expressed mainly in hepatocytes but is also synthesized in mononuclear phagocytes, neutrophils, and intestinal epithelial cells [5]. *SERPINA1* plays a crucial role in the maintenance of cell homeostasis through irreversibly inhibiting a variety of serine endopeptidases. Previous literature has paid much attention to the pathological process and treatment of *SERPINA1* deficiency in the liver and lung. Recently, a growing body of research has confirmed that *SERPINA1* shows a tremendous influence on multiple tumors, such as lung cancer [6], gastric cancer [7], and breast cancer [8]. However, the systematical analysis of *SERPINA1* in prognosis and TME regulation in cancers is rare.

In this study, the role of *SERPINA1* in prognosis and immunological regulation in tumors was analyzed through multiple bioinformatics approaches. The differential expressions and prognostic values of *SERPINA1* in different cancers were explored comprehensively. We also analyzed the potential connection between *SERPINA1* level and immune and molecular subtypes, biomarkers of therapeutic efficacy, and tumor-infiltrating lymphocytes. Furthermore, the role of SERPINA1A in liver hepatocellular carcinoma (LIHC) was detected using fluorescence-based multiplex immunohistochemistry (mIHC) to confirm the results we acquired. The study was designed to elucidate the potential of *SERPINA1* in tumor prognosis and immunotherapy, thus providing novel insight into the antitumor strategy.

## 2. Results

### 2.1. Pan-Cancer Differential Expressions of SERPINA1 between Tumor and Normal Tissues

**Figure 1:**
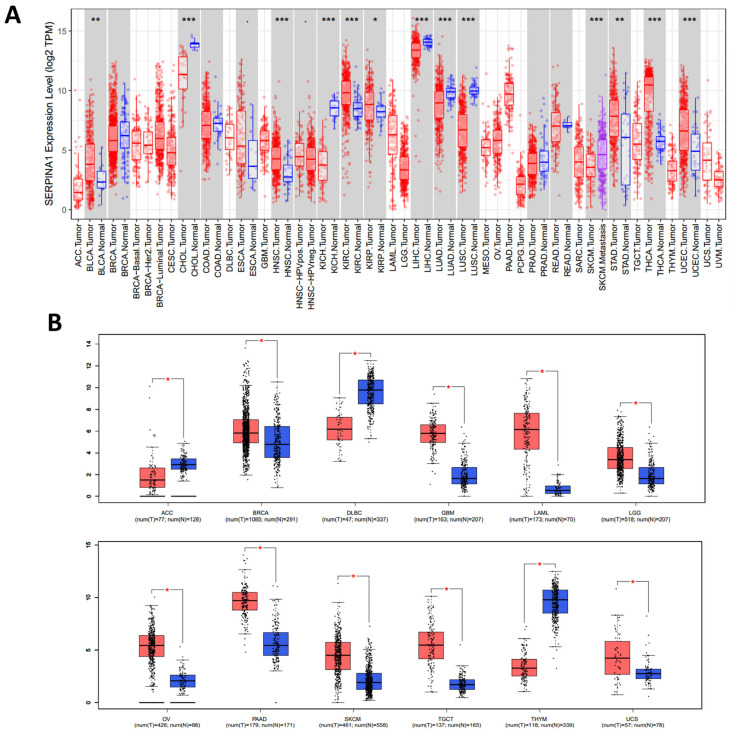
*SERPINA1* expression in human cancers. (**A**) *SERPINA1* expression in different human cancers and normal tissues according to the TIMER 2.0 database. (**B**) *SERPINA1* expression in some human cancers and normal tissues according to the GEPIA database. Red, cancer tissues. Blue, normal tissues. * *p* < 0.05, ** *p* < 0.01, *** *p* < 0.001.

The TIMER database confirmed that *SERPINA1* mRNA expression was significantly higher in BLCA (bladder urothelial carcinoma), HNSC (head and neck squamous cell carcinoma), KIRC (kidney renal clear cell carcinoma), KIRP (kidney renal papillary cell carcinoma), STAD (stomach adenocarcinoma), THCA (thyroid carcinoma), and UCEC (uterine corpus endometrial carcinoma) than in paired normal tissues. Meanwhile, *SERPINA1* expression was significantly lower in CHOL (cholangiocarcinoma), LIHC, LUAD (lung adenocarcinoma), and LUSC (lung squamous cell carcinoma) (Figure 1A). The GEPIA database was utilized to replenish the analysis of cancers without corresponding normal tissues, and the results showed that *SERPINA1* expression was overexpressed in most cancers except ACC (adrenocortical carcinoma), DLBC (lymphoid neoplasm diffuse large B-cell lymphoma), and THYM (thymoma) (Figure 1B).

**Figure 2:**
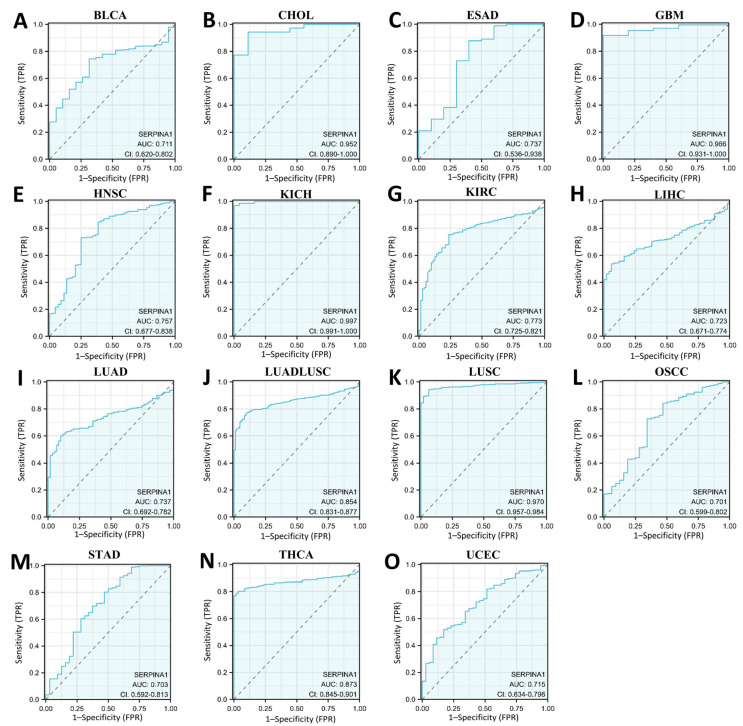
The ROC curves of *SERPINA1*: (**A**) in BLCA; (**B**) in CHOL; (**C**) in ESAD; (**D**) in GBM; (**E**) in HNSC; (**F**) in KICH; (**G**) in KIRC; (**H**) in LIHC; (**I**) in LUAD; (**J**) in LUADLUSC; (**K**) in LUSC; (**L**) in OSCC; (**M**) in STAD; (**N**) in THCA; (**O**) in UCEC.

In addition, we detected the diagnostic value of *SERPINA1* to differentiate tumors from normal tissue via the ROC curve (Figure 2). *SERPINA1* had the potential to work as a diagnostic marker in BLCA (AUC = 0.711), CHOL (AUC = 0.952), ESAD (esophagus adenocarcinoma, AUC = 0.737), GBM (glioblastoma multiforme, AUC = 0.966), HNSC (AUC = 0.757), KICH (kidney chromophobe, AUC = 0.997), KIRC (AUC = 0.773), LIHC (AUC = 0.723), LUAD (AUC = 0.737), LUADLUSC (lung adenocarcinoma and lung squamous cell carcinoma, AUC = 0.854), LUSC (AUC = 0.984), OSCC (oral squamous cell carcinoma, AUC = 0.701), STAD (AUC = 0.703), THCA (AUC = 0.873), and UCEC (AUC = 0.715).

### 2.2. Dual Prognostic Role of SERPINA1 in Human Cancers

**Figure 3:**
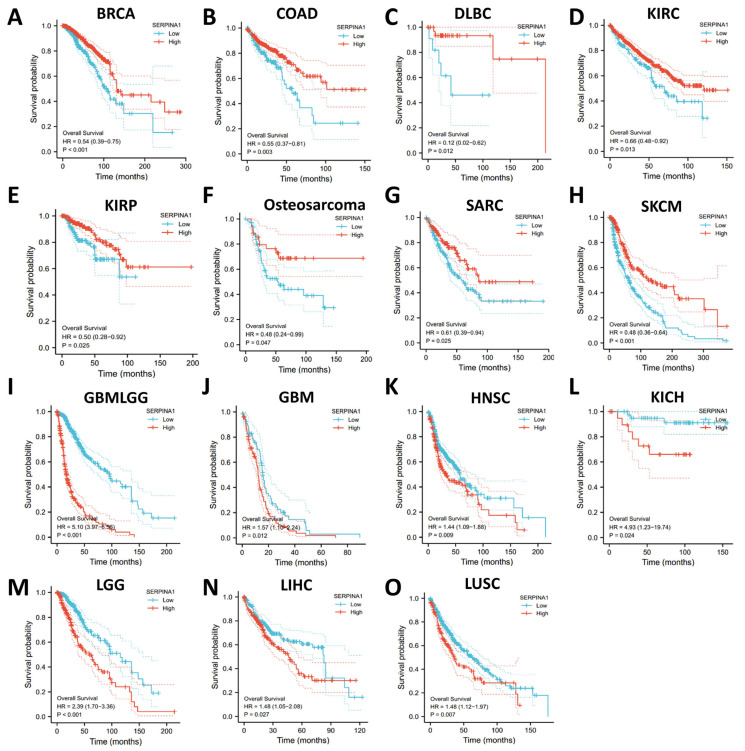
The overall survival curves of human cancers with different *SERPINA1* expressions: (**A**) in BRCA; (**B**) in COAD; (**C**) in DLBC; (**D**) in KIRC; (**E**) in KIRP; (**F**) in Osteosarcoma; (**G**) in SARC; (**H**) in SKCM; (**I**) in GBMLGG; (**J**) in GBM; (**K**) in HNSC; (**L**) in KICH; (**M**) in LGG; (**N**) in LIHC; (**O**) in LUSC.

The prognostic role of *SERPINA1* was identified using different databases. In the Xiantao database, higher *SERPINA1* expression was connected to better overall survival (OS) in BRCA (breast invasive carcinoma), COAD (colon adenocarcinoma), DLBC, KIRC, KIRP, osteosarcoma, SARC (sarcoma), and SKCM (skin cutaneous melanoma) (Figure 3A–H). However, higher *SERPINA1* expression was associated with poorer OS in GBMLGG (glioma), GBM, HNSC, KICH, LGG (brain lower-grade glioma), LIHC, and LUSC (Figure 3I–O). Furthermore, *SERPINA1* expression in most of these cancers was also related to disease-specific survival (DSS) (Figure S1). In the Kaplan–Meier plotter database, higher *SERPINA1* expression was connected to better OS in CESC (cervical squamous cell carcinoma and endocervical adenocarcinoma), THCA, and UCEC. In comparison, higher *SERPINA1* expression was associated with poorer OS in ESCA (esophageal carcinoma), TGCT (testicular germ cell tumors), and THYM (Figure S2). These results suggested that *SERPINA1* expression had the potential to be a prognostic biomarker in various cancers.

### 2.3. Associations between SERPINA1 and Immune and Molecular Subtypes

**Figure 4:**
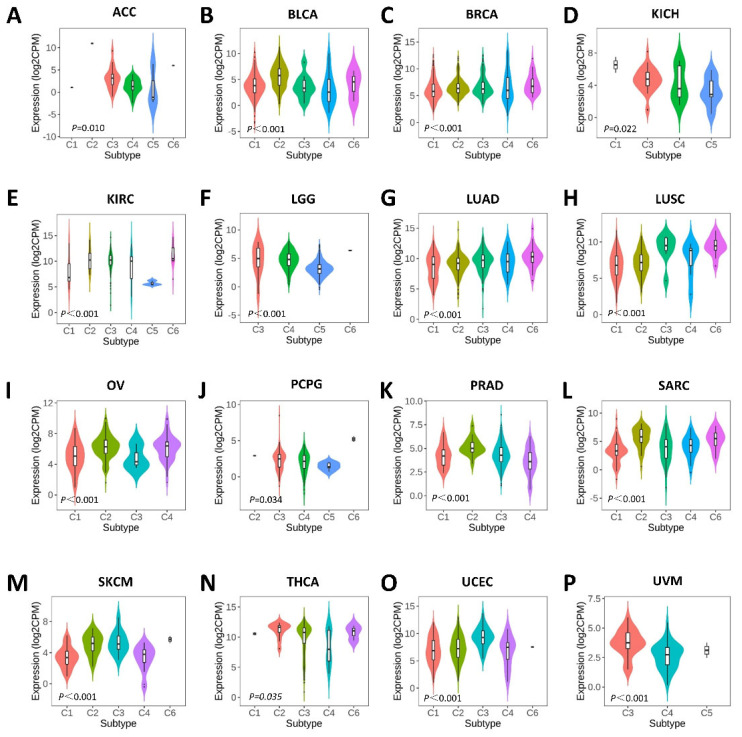
The *SERPINA1* expressions in different immune subtypes: (**A**) in ACC; (**B**) in BLCA; (**C**) in BRCA; (**D**) in KICH; (**E**) in KIRC; (**F**) in LGG; (**G**) in LUAD; (**H**) in LUSC; (**I**) in OV; (**J**) in PCPG; (**K**) in PRAD; (**L**) in SARC; (**M**) in SKMC; (**N**) in THCA; (**O**) in UCEC; (**P**) in UVM. C1, wound healing. C2, IFN-gamma dominant. C3, inflammatory. C4, lymphocyte depleted. C5, immunologically quiet. C6, TGF-β dominant.

**Figure 5:**
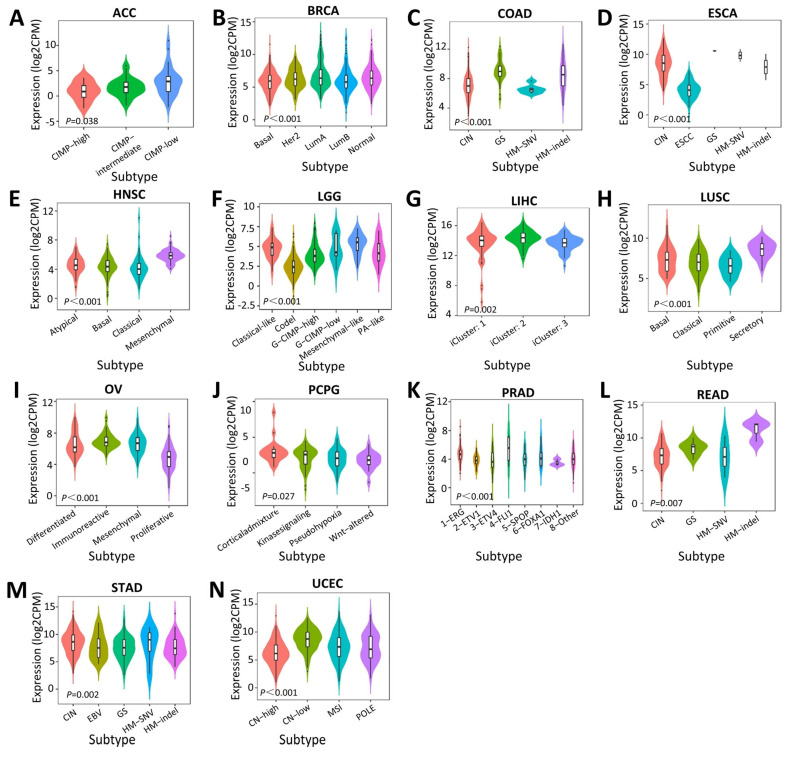
The *SERPINA1* expressions in different molecular subtypes: (**A**) in ACC; (**B**) in BRCA; (**C**) in COAD; (**D**) in ESCA; (**E**) in HNSC; (**F**) in LGG; (**G**) in LIHC; (**H**) in LUSC; (**I**) in OV; (**J**) in PCPG; (**K**) in PRAD; (**L**) in READ; (**M**) in STAD; (**N**) in UCEC.

Then, we explored the influence of *SERPINA1* on immune and molecular subtypes in human tumors through the TISIDB web portal. *SERPINA1* expression was confirmed to be associated with immune subtypes in ACC, BLCA, BRCA, KICH, KIRC, LGG, LUAD, LUSC, OV (ovarian serous cystadenocarcinoma), PCPG (pheochromocytoma and paraganglioma), PRAD (prostate adenocarcinoma), SARC, SKCM, THCA, UCEC, and UVM (uveal melanoma) (Figure 4). Different molecular subtypes showed different *SERPINA1* expressions in ACC, BRCA, COAD, ESCA, HNSC, LGG, LIHC, LUSC, OV, PCPG, PRAD, READ, STAD, and UCEC (Figure 5). The results above demonstrated that *SERPINA1* expression is closely related to the immune and molecular subtypes of various cancers.

### 2.4. Pan-Cancer Relationships between AERPINA1 and Immune Checkpoint (ICP) Genes

**Figure 6:**
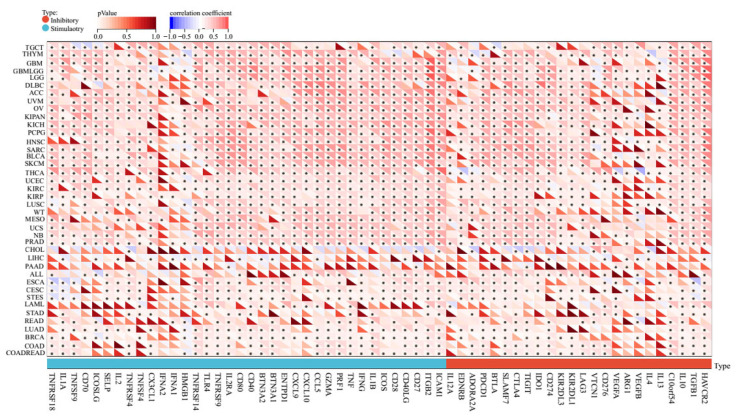
Pan-cancer association between *SERPINA1* expression and ICP genes.

ICP blockades have shown unprecedented advances in tumor immunotherapy [9]. To explore the potential role of *SERPINA1* in immunotherapy, we analyzed the relationship between *SERPINA1* expression and ICP genes, which significantly affect immune cell infiltration, through the SangerBox database [10]. *SERPINA1* expression is positively related to ICP genes in most cancer types, especially GBMLGG, OV, KIPAN (Pan-kidney cohort), and PRAD, in which more than 90% of 60 ICP genes were connected to *SERPINA1* expression (Figure 6). These results indicated that high *SERPINA1* expression might forecast the satisfactory outcome of immunotherapy targeting ICP genes, and *SERPINA1* might be a novel immunotherapy target for its influence on ICP genes. Furthermore, *SERPINA1* related to a few ICP genes only in CHOL and PAAD, which suggested that corresponding patients with high *SERPINA1* expression might respond poorly to immunotherapy referring to ICP genes. Based on the findings above, we considered *SERPINA1* a potential prognostic biomarker or a novel therapeutic target for immunotherapy in human cancers.

### 2.5. Connections between SERPINA1 and Tumor Mutational Burden (TMB), Microsatellite Instability (MSI), and Estimation of Stromal and Immune Cells in Malignant Tumor Tissues Using Expression Data (ESTIMATE)

**Figure 7:**
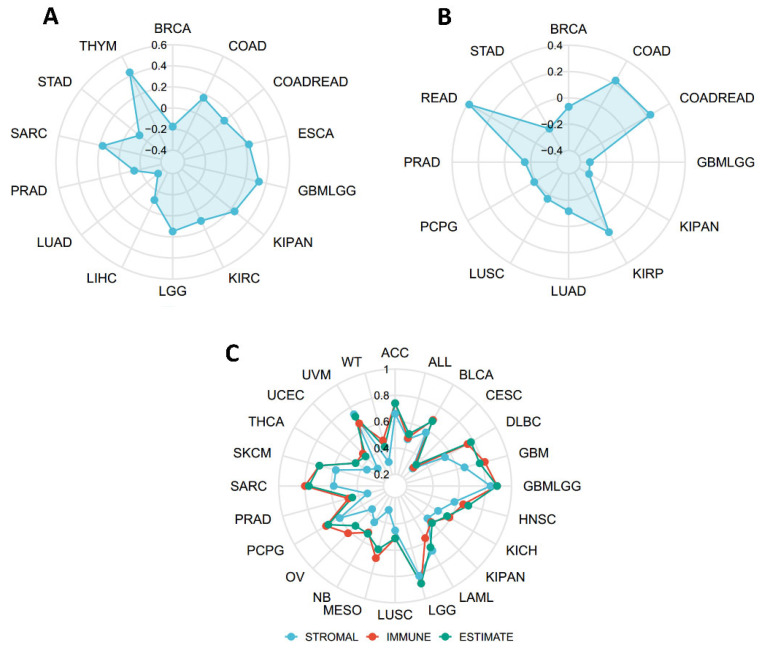
The connection between *SERPINA1* expression and biomarkers of therapeutic efficacy in human cancers. (**A**) The connection between *SERPINA1* and TMB. (**B**) The connection between *SERPINA1* and MSI. (**C**) The connection between *SERPINA1* and ESTIMATE score.

To determine the immune value of *SERPINA1* in the tumor microenvironment (TME), we explored the connections between *SERPINA1* expression and TMB and MSI, which have been confirmed to influence immunotherapy efficacy significantly [11,12]. Results analyzed using the SangerBox database demonstrated that *SERPINA1* expression was positively associated with TMB in COAD, COADREAD, ESCA, GBMLGG, KIPAN, KIRC, LGG, SARC, and THYM and negatively connected to TMB in BRCA, LIHC, LUAD, PRAD, and STAD (Figure 7A). As for MSI and *SERPINA1* expression, there were positive associations in COAD, COADREAD, KIRP, and READ, and negative correlations in BRCA, GBMLGG, KIRPAN, LUAD, LUSC, PCPG, PRAD, and STAD (Figure 7B). Subsequently, we detected a relationship between *SERPINA1* and three scores of ESTIMATE. The result confirmed significant positive associations between *SERPINA1* expression and all three scores in ACC, ALL, BLCA, CESC, DLBC, GBM, GBMLGG, HNSC, KICH, KIPAN, LAML, LGG, LUSC, MESO (mesothelioma), NB (neuroblastoma), OV, PCPG, PRAD, SARC, SKMC, THCA, UCEC, UVM, and WT (high-risk Wilms tumor) (Figure 7C). These results suggested that *SERPINA1* may play a vital role in antitumor immunity via affecting the composition of the TME.

### 2.6. Correlations between SERPINA1 and Immune Cell Infiltration in the TME

**Figure 8:**
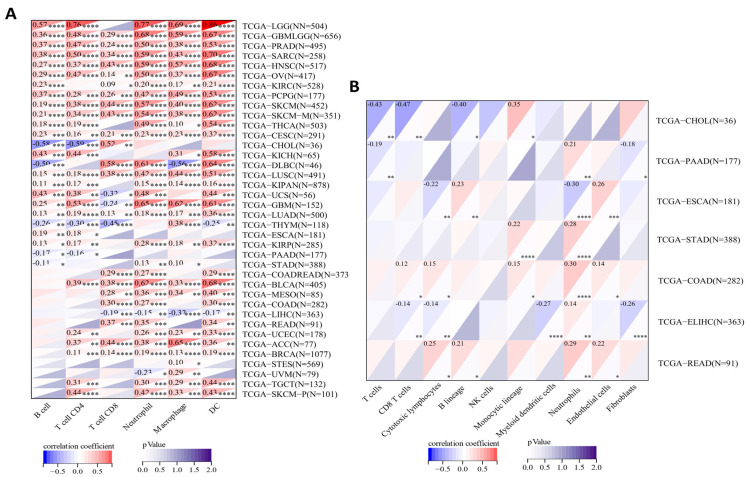
The relationship between *SERPINA1* expression and tumor-infiltrating immune cells: (**A**) in pan-cancer; (**B**) in seven digestive cancers. * *p* < 0.05, ** *p* < 0.01, *** *p* < 0.001, **** *p* < 0.0001.

As mentioned above, different immune subtypes of various cancers showed different *SERPINA1* expressions. We further explored the correlations between *SERPINA1* expression and immune cells in the TME through the Timer 2.0 website. The result demonstrated that *SERPINA1* expression presented strong connections to B cells in 25 cancer types, CD4+ T cells in 28 cancer types, CD8+ T cells in 25 cancer types, neutrophils in 32 cancer types, macrophages in 31 cancer types, and dendritic cells (DCs) in 32 cancer types (Figure 8A).

Then, we focused on the relationships between *SERPINA1* expression and immune cells in seven digestive system tumors using the R package MCPcounter. The results confirmed that *SERPINA1* expression was associated with T cells, CD8+ T cells, the B lineage, and the monocytic lineage in CHOL; T cells, neutrophils, and fibroblasts in PAAD; cytotoxic lymphocytes, the B lineage, neutrophils, and endothelial cells in ESCA; the monocytic lineage and neutrophils in STAD; CD8+ T cells, cytotoxic lymphocytes, the monocytic lineage, neutrophils, and endothelial cells in COAD; CD8+ T cells, cytotoxic lymphocytes, myeloid dendritic cells, neutrophils, and fibroblasts in LIHC; cytotoxic lymphocytes, the B lineage, neutrophils, and endothelial cells in READ (Figure 8B). These findings strongly indicated the influential role of *SERPINA1* expression in digestive cancers.

Finally, we analyzed the relationship between *SERPINA1* expression and different gene markers of immune cells via the TIMER database. *SERPINA1* expression was found to relate to CD8+ T cells, macrophages, DCs, and Th17 cells in digestive cancers (Table S1). For example, *SERPINA1* showed close connections to CD8A in CD8+ T cells, CD68 in tumor-associated macrophages, IRF6 in M1 macrophages, NRP1 in DCs, STAT3 and IL17A in Th17 in most digestive cancers. ESCA (*n* = 184) and LIHC (*n* = 371) were examples to illustrate the potential immune value of *SERPINA1*. As shown in Table 1, *SERPINA1* had a solid connection to all enrolled markers of CD8+ T cells, general T Cells, B cells, M2 macrophages, neutrophils, and Treg cells in ESCA. *SERPINA1* in LIHC also showed a good relationship with most gene markers in neutrophils and natural killer (NK) cells. These results further suggested that *SERPINA1* might be vital in regulating immune cell infiltration.

### 2.7. Alteration of SERPINA1 Gene in Different Subgroups of Digestive Carcinoma

**Figure 9:**
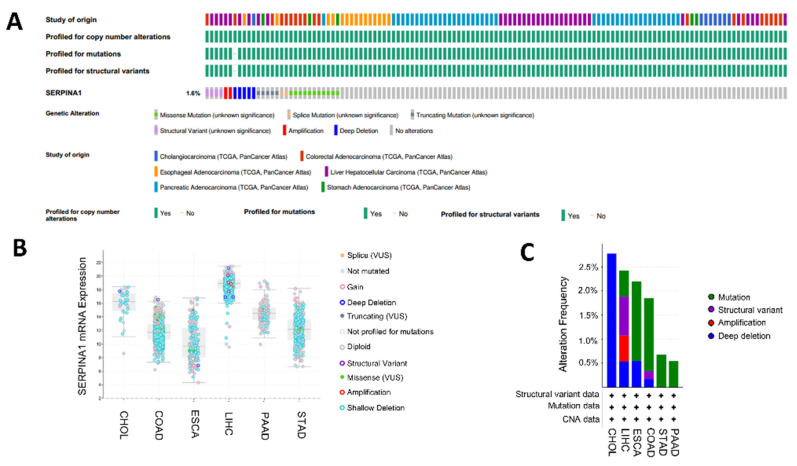
Genomic alterations of *SERPINA1* in different subgroups of digestive carcinoma. (**A**) OncoPrint of *SERPINA2* gene alterations in cancer cohort. (**B**) The main types of *SERPINA1* alterations in digestive cancers. (**C**) Details of *SERPINA1* alterations in digestive cancers.

Genomic alteration of *SERPINA1* was explored in digestive cancers except for READ, which was absent on the cBioPortal website. The result proved that the incidence of *SERPINA1* gene alteration was 1.6% (Figure 9A). Various types of *SERPINA1* gene alterations indeed led to variations in *SERPINA1* expression (Figure 9B). Furthermore, copy number variation (CNV) showed a relatively lower frequency in STAD and PAAD (Figure 9C). Then, we analyzed the associations between *SERPINA* and clinicopathologic features in seven digestive cancers via the clinical data from the TCGA database. For example, *SERPINA1* expression was significantly connected to adjacent inflammation, tumor size, pathologic stage, and AFP level in LIHC (Table S2). The relationship between *SERPINA1* and clinical features in the other six cancers is presented in Tables S3–S8. There was a weak correlation between *SERPINA1* expression and characteristics in STAD and PAAD, which were accompanied by fewer CNVs. The results above suggested that genomic alteration of *SERPINA1* occurs in cancers, and differential *SERPINA1* expression might regulate cancer progression.

### 2.8. The Influence of SERPINA1 Protein on Clinicopathologic Features and Immune Infiltrates in LIHC

**Figure 10:**
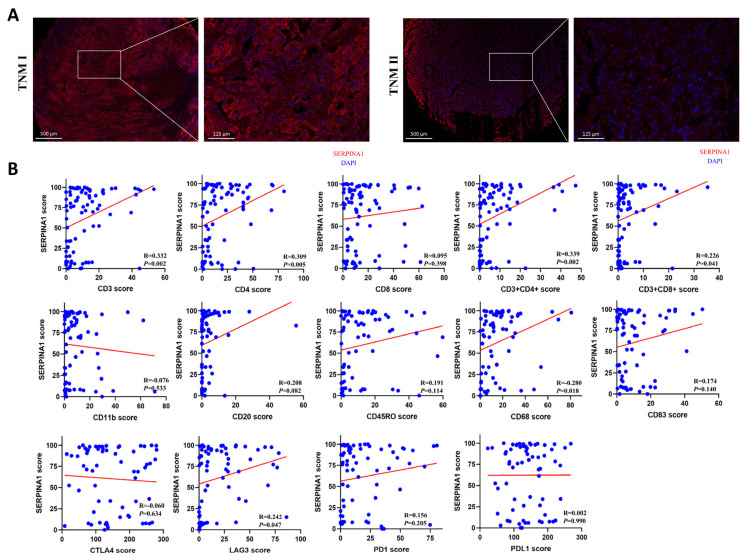
The relationships between SERPINA1 protein, clinicopathologic features, and immune infiltrates in LIHC. (**A**) The protein expression of SERPINA1 in LIHC tissues with different TNM stages. (**B**) The connection between SERPINA1 and biomarkers of immune cells. Blue dot, LIHC tissue sample. Red line, a line with linear regression.

We detected SERPINA1 protein expression in 86 LIHC tissues through fluorescence-based mIHC to confirm the results above. SERPINA1 protein was detected in the cytoplasm and mesenchyme (Figure 10A). SERPINA protein expression was significantly related to vascular invasion, tumor size, and TNM stage (Table 2). Then, we analyzed the association between the protein level of SERPINA1 and biomarkers of some immune cells in these LIHC tissues. The results revealed that SERPINA1 was significantly connected to CD3 (*p* = 0.002), CD4 (*p* = 0.005), CD3 + CD4+ (*p* = 0.002), CD3 + CD8+ (*p* = 0.041), CD68 (*p* = 0.018), and LAG3 (*p* = 0.047) (Figure 10B), which meant SERPINA1 protein expression was positively associated with T helper (Th) cells, cytotoxic lymphocytes (CTLs), and macrophages.

## 3. Discussion

An inflammatory microenvironment is a prerequisite and promoter for virtually all cancers. A growing body of research shows that inflammation greatly influences the TME composition, especially on the plasticity of cancer cells and stromal cells [13]. Tumorigenic inflammation blocks anti-tumor immunity and exerts direct pro-tumor signaling on cancer cells [14]. As a serine protease inhibitor, SERPINA1 protein (A1AT) is well known for its anti-inflammatory effect, probably attributed to the inhibition on protease which is an inflammatory vital driver [15]. Furthermore, SERPINA1 protein (A1AT) can inhibit pro-inflammatory cytokine release in monocytes via the NFκB pathway [16]. However, SERPINA1 protein (A1AT) was also confirmed to activate leukocytes and play an inflammatory role [17]. In addition, SERPINA1 protein (A1AT) is inclined to promote the production of Tregs, decrease lymphocyte infiltration, and inhibit the differentiation and maturation of DCs [18,19,20]. Cancer cells can destroy surrounding tissues through releasing elastase, plasmin, and cathepsin, then spread locally, while SERPINA1 protein (A1AT) can inactivate these enzymes. These studies suggested that *SERPINA1* has the potential to influence the TME and might be a promising biomarker for immunotherapy, but the role of SERPINA1 in TME regulation and cancer prognosis is unclear.

In this study, we first explored the differential expressions of *SERPINA1* in tumor and normal tissues through multiple databases. Previous studies have demonstrated that *SERPINA1* was overexpressed in pancreatic and breast cancer tissues but downregulated in lung cancer [21,22,23]. Our findings showed that *SERPINA1* was upregulated in most cancer tissues except CHOL, LIHC, LUAD, LUSC, ACC, DLBC, and THYM. These results suggested that SERPINA1 may play a different role in different cancers.

Previous research confirmed serum SERPINA1 as a potential biomarker for cancer diagnosis and prognosis [22,24,25]. However, serum SERPINA1 may show little association with *SERPINA1* expression in tissues [23]. Then, the association between *SERPINA1* expression in cancer tissues and the prognosis was analyzed. Our results, obtained from the Xiantao web tool and Kaplan–Meier plotter database, demonstrated that *SERPINA1* expression in tissues had a significant prognostic value for many cancers. Next, we detected the relationship between *SERPINA1* expression and immune subtypes and molecular subtypes of human cancers via the TIMER database and the R package. The results confirmed that *SERPINA1* expressions in different immune subtypes and molecular subtypes of most cancers were significantly different, indicating that *SERPINA1* may affect the immune microenvironment and cancer prognosis. Moreover, we confirmed that *SERPINA1* expression was significantly associated with clinical features in seven digestive cancers, similar to a paper proving that different *SERPINA1* levels exist in patients with varying characteristics in colorectal cancer [26].

ICPs are inhibitory receptors expressed on T cells or other immune cells, and tumors can upregulate ICPs to achieve immune escape [27]. Therefore, the expression of ICPs in the TME can influence the clinical efficacy of immune checkpoint blockade (ICB) treatment. Our results based on the SangerBox database demonstrated that *SERPINA1* expression was significantly connected to ICP genes in most cancer types, especially GBMLGG, OV, KIPAN, and PRAD, which suggested that patients with these cancers may respond well to ICB treatment. Patients with higher TMB can generate a more significant neoantigen load and induce a more robust neoantigen-specific T cell response, suggesting that patients benefit more from ICB treatment [28]. MSI is caused by functional defects in DNA mismatch repair, and patients with high MSI scores have better immunotherapy outcomes [29]. Our results found that *SERPINA1* expression was associated with TMB and MSI in some cancers, positively or negatively, indicating that *SERPINA1* plays a different role in the prediction of immunotherapy effect in various human cancers. Above results suggested that *SERPINA1* might be important in cancer immunotherapy.

The ESTIMATE score is utilized to speculate the proportion of stromal and immune cells in the TME [30]. Through the SangerBox database, we demonstrated that *SERPINA1* was positively connected to immune scores, stromal scores, and ESTIMATE scores in many cancers. Tumor-infiltrating lymphocytes (TILs) have an outsized influence on cancer prognosis and immune therapy [31,32]. This study confirmed that *SERPINA1* strongly connects to TILs, which means a high probability of regulating the TME. For example, *SERPINA1* expression was closely related to CD4+ T cells, CD8+ T cells, neutrophils, and macrophages in most cancer types. Particularly in ESCA, *SERPINA1* was connected to PDCD1 and CTLA4, which are classical targets of immunotherapy [33,34]. Furthermore, *SERPINA1* demonstrated significant association with CD8+ T cells, general T cells, B cells, M2 macrophages, neutrophils, and Treg cells in ESCA. These results suggested that SERPINA1 might affect immunotherapy efficiency through regulating TIL compositions in the TME.

Finally, we explored the SERPINA1 protein (A1AT) in LIHC tissues via fluorescence-based mIHC to preliminarily prove the results acquired. Similar to the findings regarding the SERPINA1 gene, SERPINA1 protein (A1AT) in LIHC tissues was associated with clinical features. In addition, the positive relationship between SERPINA1 level and some biomarkers of immune cells suggested the SERPINA1 protein (A1AT) might affect the infiltrations of Th cells, CTLs, and macrophages, as well as the efficacy of immunotherapy targeting LAG3.

Although we performed a systematic analysis on the role of *SERPINA1* in pan-cancer, and cross-certified the result through different databases and R package, the study has some limitations. First, systematic bias may be caused by the difference in RNA-seq data from different databases. Second, the function of the SERPINA1 protein should be verified *in vitro* and *in vivo*. Third, we preliminarily deduced that *SERPINA1* had a close connection to the immune microenvironment, but there was no direct evidence to prove the influence of *SERPINA1* on immunotherapy efficiency and prognosis. In the future, more investigations are needed to certify the prognostic and immunological value of *SERPINA1* and explore the underlying mechanism.

## 4. Materials and Methods

### 4.1. Differential Expression Analysis

The TIMER 2.0 tool (http://timer.cistrome.org/ (accessed on 15 March 2022)) and the GEPIA database (http://gepia.cancer-pku.cn/ (accessed on15 March 2022)) were utilized to compare the differential expressions of *SERPINA1* in various cancer tissues and normal tissues [35,36].

### 4.2. Survival Analysis

The relationships between *SERPINA1* expression and OS or DSS were analyzed using the Xiantao web tool (https://www.xiantao.love/ (accessed on 15 March 2022)) and Kaplan–Meier plotter database (https://kmplot.com/ (accessed on 15 March 2022)) [37,38].

### 4.3. Analysis of SERPINA1 Expression and Immune and Molecular Subtypes

The TISIDB web portal (http://cis.hku.hk/TISIDB/ (accessed on 16 March 2022)) was used to detect the associations between *SERPINA1* expression and immune or molecular subtypes of different cancers [39].

### 4.4. Analysis of SERPINA1 Expression and Biomarkers of Therapeutic Efficacy

To explore the relationships between *SERPINA1* expression and ICP genes, TMB, MSI, and ESTIMATE, the SangerBox database (http://sangerbox.com/ (accessed on 20 March 2022)) was utilized comprehensively [10].

### 4.5. Analysis of SERPINA1 Expression and Immune Cell Infiltration

The associations between *SERPINA1* level and six immune cells were explored in pan-cancers through the Timer 2.0 website. Then, we analyzed the connections between *SERPINA1* expression and the absolute abundances of 8 immune cells and two stromal cells in seven digestive system tumors using the R package MCPcounter. The relationships between *SERPINA1* expression and marker genes of immune cells were explored via the TIMER database.

### 4.6. Analysis of SERPINA1 Genomic Alterations

The cBioPortal for Cancer Genomics (https://docs.cbioportal.org/ (accessed on 10 April 2022)) was used to confirm the genomic alterations of *SERPINA1* in digestive system tumors.

### 4.7. Analysis of SERPINA1 Expression and Clinicopathologic Features

The RNAseq data of seven digestive cancers were downloaded from the TCGA database (https://portal.gdc.cancer.gov (accessed on 10 April 2022)). Data without clinical information were discarded, and the R package STATS was utilized to analyze the associations between *SERPINA1* expression and clinicopathologic features in each digestive cancer.

### 4.8. Tissue Samples and Patient Data

All hepatic tissue samples were collected from tumor immunotherapy LIHC patients admitted to the First Affiliated Hospital of Nantong University. A total of 86 LIHC tissue samples were detected in this study. None of the patients received any chemotherapy or radiation before surgery or biopsy. The study was approved by the Ethics Committee of Nantong University, and informed consent was provided by patients or their guardians.

### 4.9. Fluorescence-Based mIHC

H&E sections were observed for each tissue sample to determine the correct site. An array (12 × 10) was designed on the blank wax block, and the Quick Ray Master UATM-272A (UNITMA, Seoul, Republic of Korea) was used to drill holes into the receptor module. Tissue columns with a diameter of 1.5 mm were taken out from donor tissue blocks and then inserted into corresponding holes in receptor blocks. The receptor blocks were cut into slices with a thickness of 3 μm and placed on polylysine-coated glass slides. The slices were dewaxed and hydrated, boiled in 0.01 mM citrate buffer (pH 6.0) to retrieve antigens, and incubated with 5% goat serum to block the nonspecific site. The nuclei were stained with DAPI (#C1005, Beyotime, Shanghai, China). Then, the sections were incubated with anti-alpha 1 Antitrypsin antibody (1:200, #ab207303, Abcam, Cambridge, MA, USA) at 4 °C overnight. Opal polymer HRP Ms + Rb (#ARH1001EA, Perkin Elmer, Waltham, MA, USA) was added to the slices and incubated at dark for 3 h. Fluoroshield with DAPI (#F6057, Sigma, St. Louis, MO, USA) was used to stain nuclei and seal the sections.

### 4.10. Statistical Analysis

The experimental data of mIHC were statistically analyzed using IBM SPSS Statistics v26 (Endicott, New York, NY, USA). The correlation between SERPINA1 protein expression and clinicopathologic features was investigated using the Pearson Chi-square test. *p* < 0.05 was considered statistically significant.

## 5. Conclusions

Above all, *SERPINA1* shows potential value for the diagnosis and prognosis of many human cancers and may be involved in the immune regulation of the TME. Further study about *SERPINA1* is necessary for cancer immunotherapy and prognosis.

## Figures and Tables

**Table 1:** Relationship between *SERPINA1* and related markers of immune cells in ESCA and LIHC.

Description	Gene Markers	ESCA				LIHC			
		None		Purity		None		Purity	
		R	*p*	R	*p*	R	*p*	R	*p*
CD8+ T Cell	CD8A	0.229	0.002 *	0.194	0.009	0.106	0.041 *	0.011	0.834
	CD8B	0.321	<0.001 *	0.293	<0.001 *	0.069	0.184	−0.020	0.718
T Cell (general)	CD3D	0.358	<0.001 *	0.328	<0.001 *	0.144	0.006 *	0.053	0.328
	CD3E	0.321	<0.001 *	0.282	<0.001 *	0.228	<0.001 *	0.133	0.014 *
	CD2	0.384	<0.001 *	0.354	<0.001 *	0.191	<0.001 *	0.095	0.078
B Cell	CD19	0.362	<0.001 *	0.325	<0.001 *	0.082	0.114	0.006	0.905
	CD79A	0.277	<0.001 *	0.238	<0.001 *	0.218	<0.001 *	0.116	0.031 *
Monocyte	CD86	0.066	0.375	0.019	0.801	0.114	0.029 *	−0.003	0.961
	CSF1R	0.282	<0.001 *	0.257	<0.001 *	0.098	0.059	−0.002	0.680
TAM	CCL2	0.132	0.0744	0.088	0.242	0.083	0.112	−0.045	0.407
	CD68	0.258	<0.001 *	0.258	<0.001 *	0.161	0.002 *	0.069	0.199
	IL10	0.125	0.090	0.087	0.247	0.119	0.022 *	0.016	0.760
M1	NOS2	0.451	<0.001 *	0.47	<0.001 *	0.035	0.499	0.022	0.680
	IRF5	−0.286	<0.001 *	−0.317	<0.001 *	−0.109	0.036 *	−0.133	0.013 *
	PTGS2	0.06	0.417	0.043	0.571	0.195	<0.001 *	0.087	0.106
M2	CD163	0.339	<0.001 *	0.318	<0.001 *	0.161	0.002 *	0.070	0.197
	VSIG4	0.238	0.001 *	0.206	0.005 *	0.101	0.052	−0.003	0.949
	MS4A4A	0.287	<0.001 *	0.253	<0.001 *	0.172	<0.001 *	0.065	0.229
Neutrophils	CEACAM8	0.293	<0.001 *	0.274	<0.001 *	0.074	0.155	0.058	0.284
	ITGAM	0.251	<0.001 *	0.234	0.001 *	−0.063	0.226	−0.162	0.003 *
	CCR7	0.428	<0.001 *	0.401	<0.001 *	0.278	<0.001 *	0.1869	<0.001 *
NK Cell	KIR2DL1	0.123	0.094	0.094	0.210	−0.046	0.367	−0.062	0.250
	KIR2DL3	0.035	0.636	0.033	0.665	−0.061	0.240	−0.118	0.028 *
	KIR2DL4	0.078	0.29	0.05	0.501	−0.118	0.023 *	−0.163	0.002 *
	KIR3DL1	0.171	0.020 *	0.153	0.040 *	−0.063	0.023 *	−0.109	0.042 *
	KIR3DL2	−0.049	0.508	−0.092	0.221	−0.029	0.573	−0.084	0.121
	KIR2DL3	0	0.996	0	0.999	−0.061	0.240	−0.118	0.028 *
	KIR2DS4	0.042	0.571	0.041	0.583	−0.078	0.135	−0.085	0.117
Dendritic Cell	HLA-DPB1	0.4	<0.001 *	0.38	<0.001 *	0.15	0.004 *	0.055	0.307
	HLA-DQB1	0.362	<0.001 *	0.329	<0.001 *	0.11	0.034 *	0.018	0.743
	HLA-DRA	0.456	<0.001 *	0.441	<0.001 *	0.167	0.001 *	0.078	0.151
	HLA-DPA1	0.431	<0.001 *	0.414	<0.001 *	0.178	<0.001 *	0.089	0.094
	CD1C	0.063	0.393	0.002	0.977	0.217	<0.001 *	0.131	0.015 *
	NRP1	0.178	0.016 *	0.142	0.058 *	0.060	0.250	0.021	0.697
	ITGAX	0.355	<0.001 *	0.24	<0.001 *	0.126	0.015 *	0.027	0.624
Th1	TBX21	0.308	<0.001 *	0.272	<0.001 *	0.131	0.012 *	0.034	0.526
	STAT4	0.275	<0.001 *	0.237	0.001 *	0.12	0.021 *	0.043	0.422
	STAT1	0.111	0.134	0.085	0.250	0.175	<0.001 *	0.136	0.011 *
	IFNG	0.205	0.005 *	0.172	0.021 *	−0.022	0.669	−0.083	0.122
	TNF	0.066	0.373	0.046	0.537	0.155	0.003 *	0.075	0.165
	IL12A	0.201	0.006 *	0.19	0.011 *	0.008	0.884	−0.040	0.454
	IL12B	0.262	<0.001 *	0.227	0.002 *	0.131	0.012 *	0.049	0.368
Th2	GATA3	0.07	0.340	0.034	0.649	0.107	0.040 *	−0.002	0.965
	STAT6	0.278	<0.001 *	0.302	<0.001 *	−0.040	0.444	−0.050	0.352
	STAT5A	0.444	<0.001 *	0.432	<0.001 *	−0.116	0.026 *	−0.189	<0.001 *
	IL13	0.173	0.019 *	0.146	0.050	−0.099	0.057	−0.12	0.026 *
Tfh	BCL6	−0.504	<0.001 *	−0.512	<0.001 *	0.019	0.711	0.011	0.833
	IL21	0.09	0.225	0.059	0.432	0.045	0.392	0.012	0.821
Th17	STAT3	0.059	0.427	0.047	0.531	0.161	0.002 *	0.129	0.016 *
	IL17A	0.378	<0.001 *	0.379	<0.001 *	0.079	0.131	0.067	0.218
Treg	FOXP3	0.255	<0.001 *	0.222	0.003 *	0.025	0.633	−0.035	0.518
	CCR8	0.314	<0.001 *	0.282	<0.001 *	0.179	<0.001 *	0.107	0.048 *
	STAT5B	0.189	0.010 *	0.193	0.009 *	−0.022	0.677	0.013	0.814
	TGFB1	−0.508	<0.001 *	−0.574	<0.001 *	0.205	<0.001 *	0.113	0.036 *
T cell exhaustion	PDCD1	0.301	<0.001 *	0.272	<0.001 *	0.119	0.021 *	0.028	0.608
	CTLA4	0.292	<0.001 *	0.261	<0.001 *	0.114	0.028 *	0.029	0.589
	LAG3	0.143	0.052	0.106	0.158	−0.032	0.537	−0.091	0.091
	HAVCR2	0.256	<0.001 *	0.227	0.002 *	0.080	0.126	−0.047	0.380
	GZMB	0.115	0.120	0.073	0.333	−0.013	0.801	−0.093	0.085

* *p* < 0.05.

**Table 2:** SERPINA1 protein expression level and LIHC patient clinicopathological characteristics.

Characteristics	n	SERPINA1 Expression (%)		χ^2^	*p*
		Low or No	High		
Total	86	37 (43.02)	49 (56.98)		
Gender				0.054	0.816
Male	64	28 (43.75)	36 (56.25)		
Female	22	9 (40.91)	13 (59.09)		
Age				0.042	0.837
<60	76	33 (43.42)	43 (56.58)		
≥60	10	4 (40.00)	6 (60.00)		
Hepatitis B virus infection				2.447	0.118
0	10	2 (20.00)	8 (80.00)		
1	76	35 (46.05)	41 (53.95)		
Differentiation				3.046	0.218
Well	8	3 (37.50)	5 (62.50)		
Moderate	62	24 (38.71)	38 (61.29)		
Poor	16	10 (62.50)	6 (37.50)		
Vascular invasion				6.394	0.011 *
0	46	14 (30.43)	32 (69.57)		
1	40	23 (57.50)	17 (42.50)		
T				4.879	0.027 *
T1	42	13 (30.95)	29 (69.05)		
T2 + T3	44	24 (54.55)	20 (45.45)		
N				2.712	0.100
N0	84	35 (41.67)	49 (58.33)		
N1	2	2 (100.00)	0 (0.00)		
TNM stage				4.879	0.027 *
Ⅰ	42	13 (30.95)	29 (69.05)		
Ⅱ + Ⅲ	44	24 (54.55)	20 (45.45)		

* *p* < 0.05.

## Data Availability

All data generated or analyzed are included in the current manuscript and they are available from the corresponding author upon reasonable request.

## Supplementary Materials

The following supporting information can be downloaded at: https://www.mdpi.com/article/10.3390/ijms241411566/s1. Figure S1. The disease specific survival curves of human cancers with different SERPINA1 expressions. Figure S2. The overall survival curves of human cancers with different SERPINA1 expressions in the Kaplan–Meier plotter database. Table S1. The relationship between SERPINA1 expression and different gene markers of immune cells. Table S2. The relationships between SERPINA1 expression and clinical features in LIHC. Table S3. The relationships between SERPINA1 expression and clinical features in STAD. Table S4. The relationships between SERPINA1 expression and clinical features in CHOL. Table S5. The relationships between SERPINA1 expression and clinical features in COAD. Table S6. The relationships between SERPINA1 expression and clinical features in READ. Table S7. The relationships between SERPINA1 expression and clinical features in PAAD. Table S8. The relationships between SERPINA1 expression and clinical features in ESCA.

## Abbreviations

ACC	Adrenocortical carcinoma
BLCA	Bladder urothelial carcinoma
BRCA	Breast invasive carcinoma
CESC	Cervical squamous cell carcinoma and endocervical adenocarcinoma
CHOL	Cholangiocarcinoma
CNV	Copy number variation
COAD	Colon adenocarcinoma
CTL	Cytotoxic lymphocyte
DC	Dendritic cell
DLBC	Lymphoid neoplasm diffuse large B-cell lymphoma
ESAD	Esophagus adenocarcinoma
ESCA	Esophageal carcinoma
ESTIMATE	Estimation of Stromal and Immune cells in Malignant Tumor tissues using Expression data
GBM	Glioblastoma multiforme
GBMLGG	Glioma
HNSC	Head and neck squamous cell carcinoma
ICP	Immune checkpoint
KICH	Kidney chromophobe
KIPAN	Pan-kidney cohort
KIRC	Kidney renal clear cell carcinoma
KIRP	Kidney renal papillary cell carcinoma
LGG	Brain lower grade glioma
LIHC	Liver hepatocellular carcinoma
LUAD	Lung adenocarcinoma
LUADLUSC	Lung adenocarcinoma and lung squamous cell carcinoma
LUSC	Lung squamous cell carcinoma
MESO	Mesothelioma
MSI	Microsatellite instability
NB	Neuroblastoma
NK	Natural killer cell
OS	Overall survival
OSCC	Oral squamous cell carcinoma
OV	Ovarian serous cystadenocarcinoma
PCPG	Pheochromocytoma and paraganglioma
PRAD	Prostate adenocarcinoma
SARC	Sarcoma
SERPINA1	Serpin family A member 1
SKCM	Skin cutaneous melanoma
STAD	Stomach adenocarcinoma
TCGA	The Cancer Genome Atlas
TGCT	Testicular germ cell tumors
THCA	Thyroid carcinoma
THYM	Thymoma
TMB	Tumor mutational burden
TME	Tumor microenvironment
UCEC	Uterine corpus endometrial carcinoma
UVM	Uveal melanoma
WT	High-risk Wilms tumor
