# Supplementary material for: Serpin Family A Member 1 Is Prognostic and Involved in Immunological Regulation in Human Cancers
Source: Int J Mol Sci. 2023 Jul 17;24(14):11566. doi: 10.3390/ijms241411566 (PMC10380780; doi:10.3390/ijms241411566)
Supplement: Supplementary file 1 [file ijms-24-11566-s001.zip › Table S6.pdf]

Table S6 Relationship between SERPINA1 expression and clinical features in READ

Characteristic	SERPINA1 expression, n (%)		P
	Low	High	
Total	83 (50)	83 (50)	
Gender			0.533
Female	35 (46.7)	40 (53.3)	
Male	48 (52.7)	43 (47.3)	
Age			0.877
<=65	42 (51.2)	40 (48.8)	
>65	41 (48.8)	43 (51.2)	
Race			**0.018***
Asian	0 (0)	1 (100)	
Black or African American	6 (100)	0 (0)	
White	43 (53.1)	38 (46.9)	
BMI			0.224
<25	9 (45)	11 (55)	
>=25	34 (64.2)	19 (35.8)	
Residual tumor			0.561
R0	60 (49.2)	62 (50.8)	
R1	2 (100)	0 (0)	
R2	6 (50)	6 (50)	
CEA level			0.587
<=5	30 (46.2)	35 (53.8)	
>5	25 (53.2)	22 (46.8)	
Perineural invasion			0.862
NO	23 (57.5)	17 (42.5)	
YES	7 (50)	7 (50)	
Lymphatic invasion			0.757
NO	40 (47.6)	44 (52.4)	
YES	33 (51.6)	31 (48.4)	
History of colon polyps			0.065
NO	63 (54.8)	52 (45.2)	
YES	11 (34.4)	21 (65.6)	
Colon polyps present			0.925
NO	35 (56.5)	27 (43.5)	
YES	6 (50)	6 (50)	
T stage			**0.015***
T1+ T2	12 (32.4)	25 (67.6)	
T3+ T4	70 (55.1)	57 (44.9)	
N stage			0.069
N0	35 (41.7)	49 (58.3)	
N1	25 (55.6)	20 (44.4)	
N2	21 (63.6)	12 (36.4)	

M stage			0.422
M0	62 (49.2)	64 (50.8)	
M1	14 (60.9)	9 (39.1)	
Pathologic stage			**0.024***
Stage I+ II	34(36.7)	47 (63.3)	
Stage III+ IV	45 (62.7)	30 (37.3)	
