# Supplementary material for: Serpin Family A Member 1 Is Prognostic and Involved in Immunological Regulation in Human Cancers
Source: Int J Mol Sci. 2023 Jul 17;24(14):11566. doi: 10.3390/ijms241411566 (PMC10380780; doi:10.3390/ijms241411566)
Supplement: Supplementary file 1 [file ijms-24-11566-s001.zip › Table S7.pdf]

Table S7 Relationship between SERPINA1 expression and clinical features in PAAD

Characteristic	SERPINA1 expression, n (%)		P
	Low	High	
	89 (50)	89 (50)	
Gender			0.651
Female	38 (47.5)	42 (52.5)	
Male	51 (52)	47 (48)	
Age			0.548
<=65	44 (47.3)	49 (52.7)	
>65	45 (52.9)	40 (47.1)	
Race			0.490
Asian	7 (63.6)	4 (36.4)	
Black or African American	2 (33.3)	4 (66.7)	
White	77 (49)	80 (51)	
Residual tumor			0.293
R0	54 (50.5)	53 (49.5)	
R1	23 (44.2)	29 (55.8)	
R2	4 (80)	1 (20)	
Histologic grade			0.175
G1	11 (35.5)	20 (64.5)	
G2	50 (52.6)	45 (47.4)	
G3	25 (52.1)	23 (47.9)	
G4	2 (100)	0 (0)	
Anatomic neoplasm subdivision			0.857
Head of Pancreas	70 (50.7)	68 (49.3)	
Other	19 (47.5)	21 (52.5)	
Smoker			0.235
No	37 (56.9)	28 (43.1)	
Yes	36 (45.6)	43 (54.4)	
Alcohol history			0.609
No	30 (46.2)	35 (53.8)	
Yes	52 (51.5)	49 (48.5)	
History of diabetes			0.398
No	52 (48.1)	56 (51.9)	
Yes	22 (57.9)	16 (42.1)	
History of chronic pancreatitis			0.936
No	66 (51.6)	62 (48.4)	
Yes	6 (46.2)	7 (53.8)	
Family history of cancer			0.098
No	27 (57.4)	20 (42.6)	

Yes	25 (39.7)	38 (60.3)	
T stage			1.000
T1	4 (57.1)	3 (42.9)	
T2	12 (50)	12 (50)	
T3	71 (50)	71 (50)	
T4	2 (66.7)	1 (33.3)	
N stage			0.581
N0	27 (54)	23 (46)	
N1	59 (48)	64 (52)	
M stage			1.000
M0	40 (50.6)	39 (49.4)	
M1	3 (60)	2 (40)	
Pathologic stage			0.935
Stage I	11 (52.4)	10 (47.6)	
Stage II	72 (49.3)	74 (50.7)	
Stage III	2 (66.7)	1 (33.3)	
Stage IV	3 (60)	2 (40)	
