# Supplementary material for: Serpin Family A Member 1 Is Prognostic and Involved in Immunological Regulation in Human Cancers
Source: Int J Mol Sci. 2023 Jul 17;24(14):11566. doi: 10.3390/ijms241411566 (PMC10380780; doi:10.3390/ijms241411566)
Supplement: Supplementary file 1 [file ijms-24-11566-s001.zip › Table S8.pdf]

Table S8 Relationship between SERPINA1 expression and clinical features in ESCA

Characteristic	SERPINA1 expression, n (%)		P
	Low	High	
Total	81 (50)	81 (50)	
Gender			0.177
Female	15 (65.2)	8 (34.8)	
Male	66 (47.5)	73 (52.5)	
Age			**0.005***
<=60	51 (61.4)	32 (38.6)	
>60	30 (38)	49 (62)	
Race			**< 0.001***
Asian	32 (84.2)	6 (15.8)	
Black or African American	5 (83.3)	1 (16.7)	
White	38 (38)	62 (62)	
Smoker			0.716
No	26 (55.3)	21 (44.7)	
Yes	49 (50.5)	48 (49.5)	
Alcohol history			0.822
No	22 (47.8)	24 (52.2)	
Yes	58 (51.3)	55 (48.7)	
Barretts esophagus			**0.012***
No	56 (52.8)	50 (47.2)	
Yes	6 (23.1)	20 (76.9)	
Reflux history			**< 0.001***
No	52 (61.9)	32 (38.1)	
Yes	16 (30.8)	36 (69.2)	
Histological type			**< 0.001***
Adenocarcinoma	12 (15)	68 (85)	
Squamous Cell Carcinoma	69 (84.1)	13 (15.9)	
Residual tumor			0.675
R0	64 (52.9)	57 (47.1)	
R1	4 (36.4)	7 (63.6)	
R2	1 (50)	1 (50)	
Histologic grade			**< 0.001***
G1	15 (93.8)	1 (6.2)	
G2	38 (57.6)	28 (42.4)	
G3	17 (38.6)	27 (61.4)	
Tumor cental location			**< 0.001***
Distal	41 (36.3)	72 (63.7)	
Mid	34 (81)	8 (19)	
Proximal	5 (83.3)	1 (16.7)	
Columnar metaplasia			**0.016***

No	38 (54.3)	32 (45.7)	
Yes	7 (25)	21 (75)	
Columnar mucosa dysplasia			0.435
High grade	9 (36)	16 (64)	
Low grade	1 (20)	4 (80)	
Negative/no	18 (47.4)	20 (52.6)	
T stage			**0.022***
T1	11 (40.7)	16 (59.3)	
T2	27 (73)	10 (27)	
T3	36 (46.8)	41 (53.2)	
T4	2 (50)	2 (50)	
N stage			**0.015***
N0	43 (65.2)	23 (34.8)	
N1	25 (39.7)	38 (60.3)	
N2	6 (66.7)	3 (33.3)	
N3	2 (33.3)	4 (66.7)	
M stage			0.148
M0	66 (54.5)	55 (45.5)	
M1	2 (25)	6 (75)	
Pathologic stage			**0.033***
Stage I	9 (56.2)	7 (43.8)	
Stage II	44 (63.8)	25 (36.2)	
Stage III	20 (40.8)	29 (59.2)	
Stage IV	2 (25)	6 (75)	
