# Supplementary material for: Serpin Family A Member 1 Is Prognostic and Involved in Immunological Regulation in Human Cancers
Source: Int J Mol Sci. 2023 Jul 17;24(14):11566. doi: 10.3390/ijms241411566 (PMC10380780; doi:10.3390/ijms241411566)
Supplement: Supplementary file 1 [file ijms-24-11566-s001.zip › Table S2.pdf]

Table S2 Relationships between SERPINA1 expression and clinical features in LIHC

Characteristic		SERPINA1 expression, n (%)		*P*
		Low	High	
Total		187 (50.00)	187 (50.00)	
Age				0.380
	≤60	84 (47.46)	93 (52.54)	
	＞60	103 (52.55)	93 (47.45)	
Gender				0.825
	Female	59 (48.76)	62 (51.24)	
	Male	128 (50.59)	125 (49.40)	
Weight (kg)				0.914
	≤70	93 (50.54)	91 (49.46)	
	＞70	80 (49.38)	82 (50.62)	
BMI (kg/m^2)				0.954
	≤25	88 (49.72)	89 (50.28)	
	＞25	81 (50.63)	79 (49.37)	
Adjacent hepatic tissue inflammation				**0.003***
	None	70 (59.32)	48 (40.68)	
	Mild	44 (43.56)	57 (56.43)	
	Severe	4 (22.22)	14 (77.78)	
Histologic grade				0.236
	G1	34 (61.82)	21 (38.18)	
	G2	83 (46.63)	95 (53.37)	
	G3	61 (49.19)	63 (50.81)	
	G4	7 (58.33)	5 (41.67)	
Residual tumor				0.621
	R0	163 (49.85)	164 (50.15)	
	R1	7 (41.18)	10 (58.82)	
	R2	0 (0.0)	1 (100.00)	
Child-Pugh grade				0.905
	A	105 (47.95)	114 (52.05)	
	B	9 (42.86)	12 (57.14)	
	C	0 (0.0)	1 (10.00)	
T stage				**0.027***
	T1	79 (43.17)	104 (56.83)	
	T2	57 (60.00)	38 (40.00)	
	T3	45 (56.25)	35 (43.75)	
	T4	5 (38.46)	8 (61.54)	
N stage				1.000
	N0	122 (48.03)	132 (51.97)	
	N1	2 (50.00)	2 (50.00)	
M stage				0.123
	M0	132 (49.25)	136 (50.75)	

M1	0 (0.00)	4 (100.00)	
Pathologic stage			**0.009***
Stage I	76 (43.93)	97 (56.07)	
Stage II	51 (58.62)	36 (41.38)	
Stage III	47 (55.29)	38 (44.71)	
Stage IV	0 (0.00)	5 (100.00)	
Vascular invasion			0.249
No	96 (46.15)	112 (53.85)	
Yes	59 (53.64)	51 (46.36)	
AFP (ng/ml)			**0.019***
≤400	114 (53.02)	101 (46.98)	
＞400	23 (35.38)	42 (64.62)	
Albumin (g/dl)			0.472
＜3.5	30 (43.48)	39 (56.52)	
≥3.5	114 (49.35)	117 (50.65)	
Prothrombin time			1.000
≤4	100 (48.08)	108 (51.92)	
＞4	43 (48.31)	46 (51.69)	
