# Supplementary material for: Serpin Family A Member 1 Is Prognostic and Involved in Immunological Regulation in Human Cancers
Source: Int J Mol Sci. 2023 Jul 17;24(14):11566. doi: 10.3390/ijms241411566 (PMC10380780; doi:10.3390/ijms241411566)
Supplement: Supplementary file 1 [file ijms-24-11566-s001.zip › Table S3.pdf]

Table S3 Relationship between SERPINA1 expression and clinical features in STAD

Characteristic	SERPINA1 expression, n (%)		P
	Low	High	
Total	187 (49.9)	188 (50.2)	
Gender			0.564
Female	70 (52.2)	64 (47.8)	
Male	117 (48.5)	124 (51.5)	
Age			0.953
<=65	83 (50.6)	81 (49.4)	
>65	103 (49.8)	104 (50.2)	
Race			0.119
Asian	43 (58.1)	31 (41.9)	
Black or African American	3 (27.3)	8 (72.7)	
White	117 (49.2)	121 (50.8)	
Anatomic neoplasm subdivision			**0.032***
Antrum/Distal	82 (59.4)	56 (40.6)	
Cardia/Proximal	21 (43.8)	27 (56.2)	
Fundus/Body	54 (41.5)	76 (58.5)	
Gastroesophageal Junction	22 (53.7)	19 (46.3)	
Other	3 (75)	1 (25)	
Reflux history			0.675
No	94 (53.7)	81 (46.3)	
Yes	23 (59)	16 (41)	
Antireflux treatment			0.789
No	67 (47.2)	75 (52.8)	
Yes	19 (51.4)	18 (48.6)	
H pylori infection			1.000
No	76 (52.4)	69 (47.6)	
Yes	9 (50)	9 (50)	
Barretts esophagus			0.491
No	104 (53.9)	89 (46.1)	
Yes	10 (66.7)	5 (33.3)	
Histological type			0.498
Diffuse Type	36 (57.1)	27 (42.9)	
Mucinous Type	8 (42.1)	11 (57.9)	
Not Otherwise Specified	98 (47.3)	109 (52.7)	
Papillary Type	4 (80)	1 (20)	
Signet Ring Type	5 (45.5)	6 (54.5)	
Tubular Type	36 (52.2)	33 (47.8)	
Residual tumor			0.574
R0	154 (51.7)	144 (48.3)	

R1	6 (40)	9 (60)	
R2	7 (43.8)	9 (56.2)	
Histologic grade			0.072
G1	3 (30)	7 (70)	
G2	59 (43.1)	78 (56.9)	
G3	117 (53.4)	102 (46.6)	
T stage			0.325
T1	12 (63.2)	7 (36.8)	
T2	34 (42.5)	46 (57.5)	
T3	87 (51.8)	81 (48.2)	
T4	52 (52)	48 (48)	
N stage			0.287
N0	58 (52.3)	53 (47.7)	
N1	55 (56.7)	42 (43.3)	
N2	32 (42.7)	43 (57.3)	
N3	35 (47.3)	39 (52.7)	
M stage			0.988
M0	166 (50.3)	164 (49.7)	
M1	12 (48)	13 (52)	
Pathologic stage			0.560
Stage I	26 (49.1)	27 (50.9)	
Stage II	62 (55.9)	49 (44.1)	
Stage III	71 (47.3)	79 (52.7)	
Stage IV	18 (47.4)	20 (52.6)	
