# Supplementary material for: Serpin Family A Member 1 Is Prognostic and Involved in Immunological Regulation in Human Cancers
Source: Int J Mol Sci. 2023 Jul 17;24(14):11566. doi: 10.3390/ijms241411566 (PMC10380780; doi:10.3390/ijms241411566)
Supplement: Supplementary file 1 [file ijms-24-11566-s001.zip › Table S4.pdf]

Table S4 Relationship between SERPINA1 expression and clinical features in CHOL

Characteristic	SERPINA1 expression		P
	Low	High	
Total	18 (50)	18 (50)	
Gender			0.738
Female	11 (55)	9 (45)	
Male	7 (43.8)	9 (56.2)	
Age			0.181
<=65	6 (35.3)	11 (64.7)	
>65	12 (63.2)	7 (36.8)	
Race			1.000
Asian	1 (33.3)	2 (66.7)	
Black or African American	1 (50)	1 (50)	
White	16 (51.6)	15 (48.4)	
BMI			1.000
<=25	5 (50)	5 (50)	
>25	12 (48)	13 (52)	
Histological type			0.209
distal	2 (100)	0 (0)	
hilar/perihilar	3 (75)	1 (25)	
intrahepatic	13 (43.3)	17 (56.7)	
Residual tumor			0.656
R0	15 (53.6)	13 (46.4)	
R1	2 (40)	3 (60)	
Histologic grade			0.862
G1	1 (100)	0 (0)	
G2	8 (53.3)	7 (46.7)	
G3	8 (44.4)	10 (55.6)	
G4	1 (50)	1 (50)	
CA19-9 level			1.000
Abnormal	9 (56.2)	7 (43.8)	
normal	7 (50)	7 (50)	
Child-Pugh grade			0.486
A	10 (52.6)	9 (47.4)	
B	2 (100)	0 (0)	
Vascular invasion			0.164
No	17 (58.6)	12 (41.4)	
Yes	1 (20)	4 (80)	
Fibrosis ishak score			0.359
0	9 (56.2)	7 (43.8)	
1/2	6 (66.7)	3 (33.3)	
3/4	0 (0)	2 (100)	
Perineural invasion			0.674

No	15 (57.7)	11 (42.3)	
Yes	3 (42.9)	4 (57.1)	
T stage			0.812
T1	10 (52.6)	9 (47.4)	
T2	5 (41.7)	7 (58.3)	
T3	3 (60)	2 (40)	
N stage			1.000
N0	14 (53.8)	12 (46.2)	
N1	3 (60)	2 (40)	
M stage			1.000
M0	15 (53.6)	13 (46.4)	
M1	3 (60)	2 (40)	
Pathologic stage			0.657
Stage I	10 (52.6)	9 (47.4)	
Stage II	3 (33.3)	6 (66.7)	
Stage III	1 (100)	0 (0)	
Stage IV	4 (57.1)	3 (42.9)	
