# Supplementary material for: Serpin Family A Member 1 Is Prognostic and Involved in Immunological Regulation in Human Cancers
Source: Int J Mol Sci. 2023 Jul 17;24(14):11566. doi: 10.3390/ijms241411566 (PMC10380780; doi:10.3390/ijms241411566)
Supplement: Supplementary file 1 [file ijms-24-11566-s001.zip › Table S5.pdf]

Table S5 Relationship between SERPINA1 expression and clinical features in COAD

Characteristic	SERPINA1 expression, n (%)		P
	Low	High	
	239 (50)	239 (50)	
Gender			0.927
Female	114 (50.4)	112 (49.6)	
Male	125 (49.6)	127 (50.4)	
Age			0.926
<=65	96 (49.5)	98 (50.5)	
>65	143 (50.4)	141 (49.6)	
Race			0.896
Asian	6 (54.5)	5 (45.5)	
Black or African American	37 (58.7)	26 (41.3)	
White	128 (55.2)	104 (44.8)	
BMI			0.300
<25	53 (60.9)	34 (39.1)	
>=25	90 (53.3)	79 (46.7)	
Residual tumor			0.303
R0	162 (46.8)	184 (53.2)	
R1	2 (50)	2 (50)	
R2	15 (62.5)	9 (37.5)	
CEA level			**0.044***
<=5	94 (48)	102 (52)	
>5	65 (60.7)	42 (39.3)	
Perineural invasion			0.289
NO	68 (50.4)	67 (49.6)	
YES	28 (60.9)	18 (39.1)	
Lymphatic invasion			0.400
NO	127 (47.7)	139 (52.3)	
YES	88 (52.4)	80 (47.6)	
History of colon polyps			0.059
NO	140 (53.4)	122 (46.6)	
YES	63 (43.2)	83 (56.8)	
Colon polyps present			0.671
NO	89 (54.9)	73 (45.1)	
YES	51 (58.6)	36 (41.4)	
T stage			0.167
T1	4 (36.4)	7 (63.6)	
T2	37 (44.6)	46 (55.4)	
T3	161 (49.8)	162 (50.2)	
T4	37 (61.7)	23 (38.3)	
N stage			**0.033***
N0	130 (45.8)	154 (54.2)	

N1	56 (51.9)	52 (48.1)	
N2	53 (61.6)	33 (38.4)	
M stage			**0.017***
M0	163 (46.7)	186 (53.3)	
M1	42 (63.6)	24 (36.4)	
Pathologic stage			**0.024***
Stage I	33 (40.7)	48 (59.3)	
Stage II	86 (46)	101 (54)	
Stage III	71 (53.4)	62 (46.6)	
Stage IV	42 (63.6)	24 (36.4)	
