# Supplementary figures and images for: Serpin Family A Member 1 Is Prognostic and Involved in Immunological Regulation in Human Cancers
Source: Int J Mol Sci. 2023 Jul 17;24(14):11566. doi: 10.3390/ijms241411566 (PMC10380780; doi:10.3390/ijms241411566)

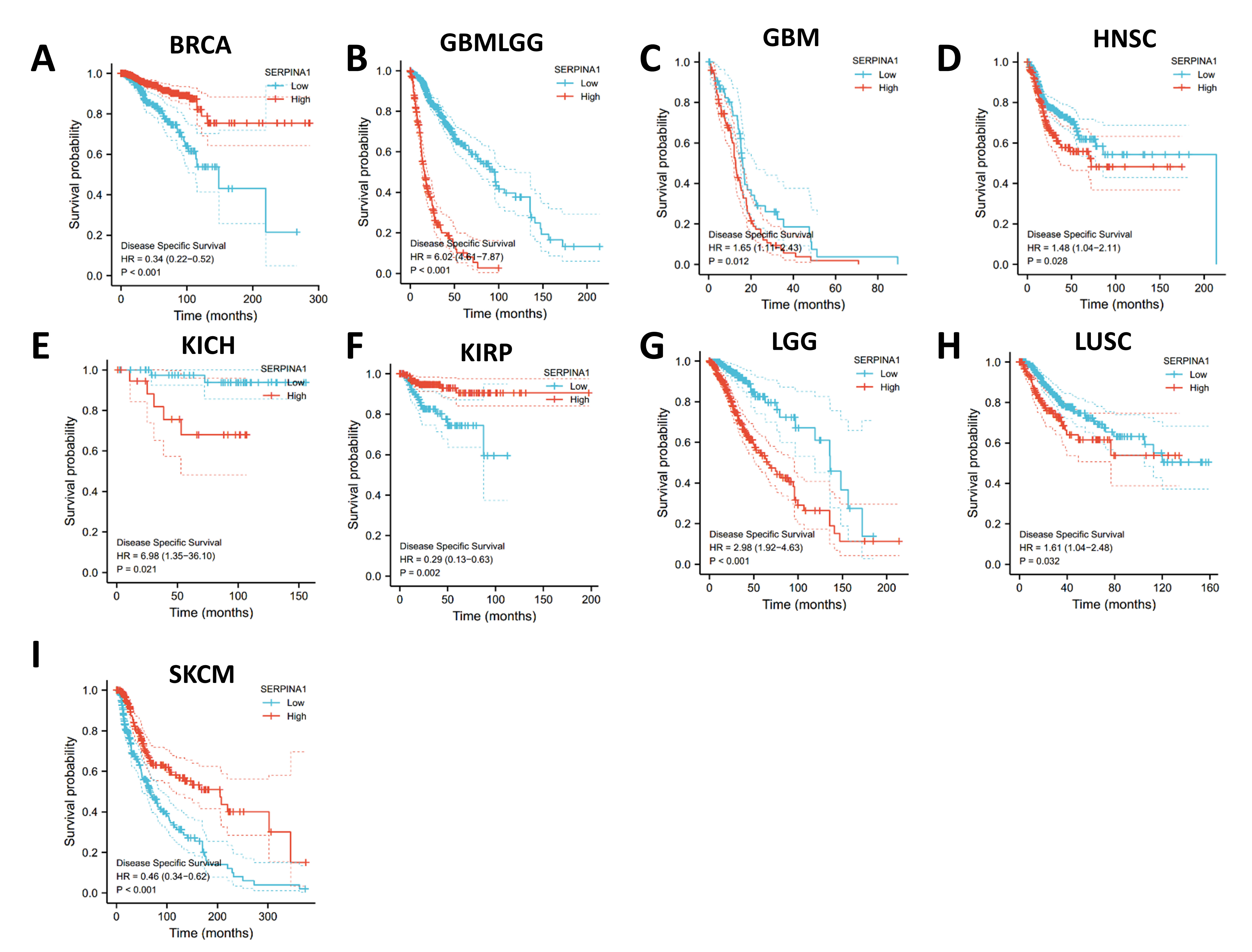

Supplement: Supplementary file 1 [file ijms-24-11566-s001.zip › Figure S1.TIF]

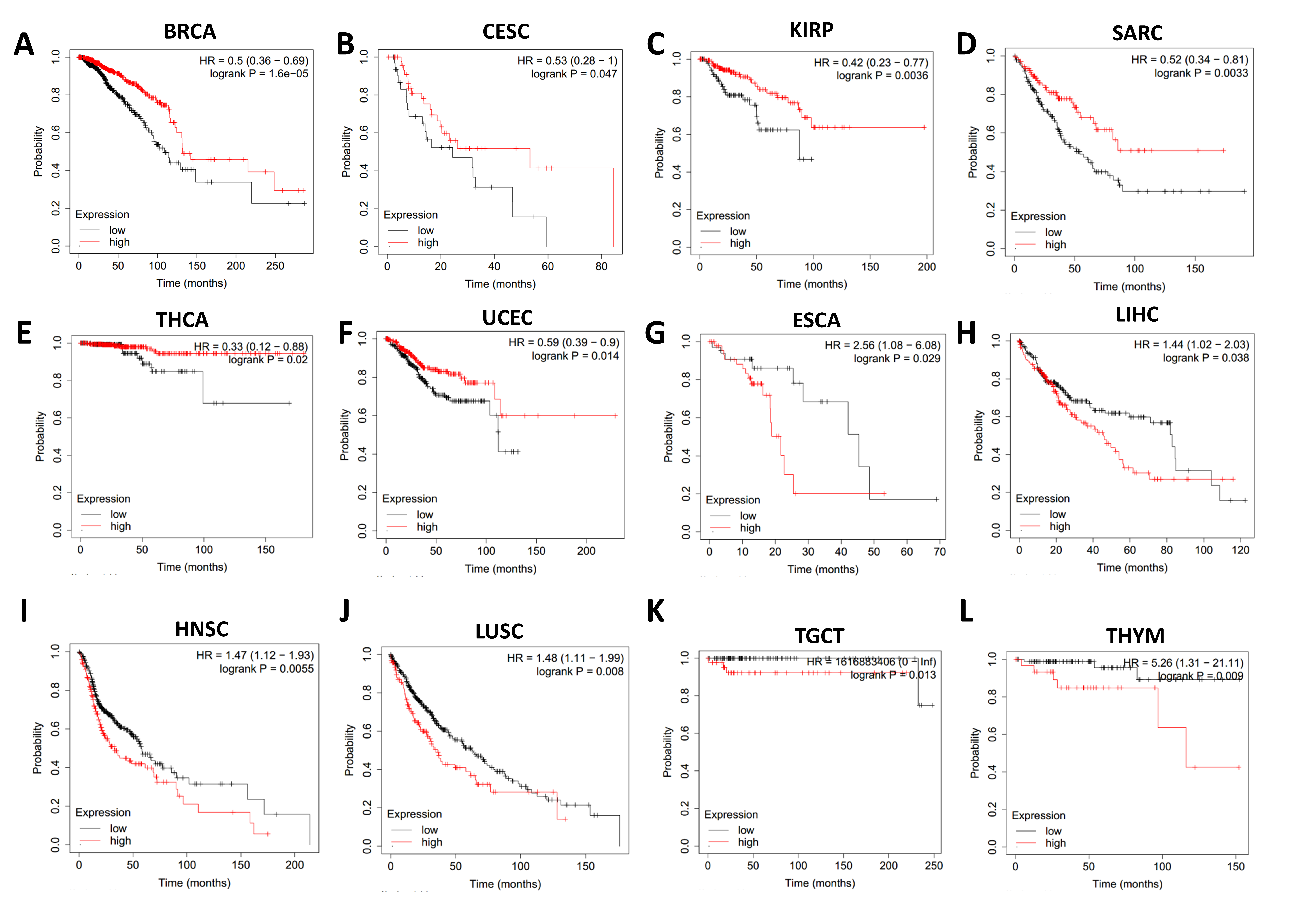

Supplement: Supplementary file 1 [file ijms-24-11566-s001.zip › Figure S2.TIF]
